# Enhanced Raman Investigation of Cell Membrane and Intracellular Compounds by 3D Plasmonic Nanoelectrode Arrays

**DOI:** 10.1002/advs.201800560

**Published:** 2018-10-23

**Authors:** Valeria Caprettini, Jian‐An Huang, Fabio Moia, Andrea Jacassi, Carlo Andrea Gonano, Nicolò Maccaferri, Rosario Capozza, Michele Dipalo, Francesco De Angelis

**Affiliations:** ^1^ Istituto Italiano di Tecnologia Via Morego 30 16163 Genoa Italy

**Keywords:** electroporation, intracellular spectroscopy, microelectrode arrays, nanopillars, permeabilization, Raman spectroscopy

## Abstract

3D nanostructures are widely exploited in cell cultures for many purposes such as controlled drug delivery, transfection, intracellular sampling, and electrical recording. However, little is known about the interaction of the cells with these substrates, and even less about the effects of electroporation on the cellular membrane and the nuclear envelope. This work exploits 3D plasmonic nanoelectrodes to study, by surface‐enhanced Raman scattering (SERS), the cell membrane dynamics on the nanostructured substrate before, during, and after electroporation. In vitro cultured cells tightly adhere on 3D plasmonic nanoelectrodes precisely in the plasmonic hot spots, making this kind of investigation possible. After electroporation, the cell membrane dynamics are studied by recording the Raman time traces of biomolecules in contact or next to the 3D plasmonic nanoelectrode. During this process, the 3D plasmonic nanoelectrodes are intracellularly coupled, thus enabling the monitoring of different molecular species, including lipids, proteins, and nucleic acids. Scanning electron microscopy cross‐section analysis evidences the possibility of nuclear membrane poration compatible with the reported Raman spectra. These findings may open a new route toward controlled intracellular sampling and intranuclear delivery of genic materials. They also show the possibility of nuclear envelope disruption which may lead to negative side effects.

## 1 Introduction

The cellular membrane is an extremely complex and dynamic environment that represents the gateway for all cell reactions and exchanges with the surrounding biological environment. Nutrients as well as molecules that drive the interactions with other cells and tissues pass through the plasma membrane. Therefore, membrane processes and mechanisms are of fundamental importance for cell functioning.^1^ In many scientific fields, gaining access to the cell interior is an essential requirement, as in the testing of new drugs, the investigation of the electrical activity within a neuronal network or the manipulation of genes to treat diseases.^2^, ^3^ Technological progress has made electroporation—that is, the application of a *transmembrane* voltage on the cell walls that transiently permeabilize the cellular membrane—an established method to gain access to the intracellular compartment.^4^, ^5^, ^6^ More recently, micro‐ and nanotechnology has intersected with 3D nanofabricated substrates and micro‐ or nanofluidic devices to improve control over the membrane poration.^7^, ^8^ After permeabilization, the cell membrane heals within a few minutes, shrinking and closing the electrically opened nanopores.^9^ However, the mechanisms of plasma membrane repair are still under investigation.^10^ In such a dynamic landscape of cells interfacing with different shapes of 3D nanostructured substrates,^11^, ^12^, ^13^, ^14^, ^15^ a deeper understanding of the cellular membrane dynamics may shed light on the yet‐unexplored behaviors of lipids, proteins, vesicles, and other constituents. In addition, it may be helpful in designing next‐generation substrates for tissue engineering and cell manipulation.^16^, ^17^ Raman microspectroscopy has been exploited to address these issues, but the large lateral resolution (1 µm) and the even larger axial resolution (7 µm) need to be improved in order to study the local behavior of the plasma membrane.^18^ Within the recent past, it has been shown that surface‐enhanced Raman spectroscopy (SERS) by means of 3D plasmonic nanostructures can provide very sensitive, localized, label‐free and noninvasive chemical analysis of living cells, enhancing the vibrational modes of molecules adsorbed onto or close to specific nanostructure hot spots. In particular, the potential of the 3D plasmonic nanostructure configuration has been shown by acquiring single Raman spectra of cells at rest in their physiological conditions.^19^, ^20^

Similar 3D nanostructured devices have been adopted to electroporate cells in vitro by using a low voltage,^21^ and the nanofluidic properties of some devices have been exploited to prove the injection of small molecules into the electroporated cells.^22^

**Figure 1:**
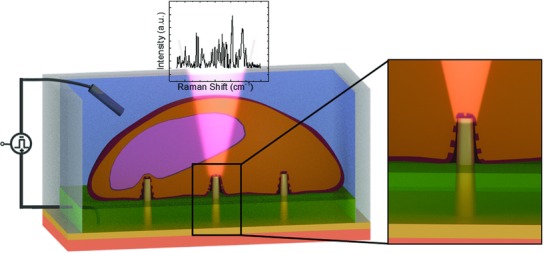
Sketch of the system with inset showing the magnification at the 3D nanostructure tip. On top of the 3D nanostructures (yellow), cells (in orange) were tightly sealed to the substrate. The plasmonic modes of the 3D nanoelectrode were excited by a 785 nm laser, and the enhanced Raman signals coming from the molecules close to it were collected. The different colors of the substrate represent bulk quartz (salmon), gold nanoelectrode (yellow), and an SU8 passivation layer (green).

In the present study, we exploit the ability of 3D vertical nanostructures combined with multielectrode arrays (MEAs) to work at the same time as nanoelectrodes for in situ electroporation and as plasmonic antennas for SERS studies of the cell membrane dynamics (see sketch in **Figure** **1**).^19^, ^20^ Using the NIH‐3T3 cell model, we investigated the cell membrane dynamics when the cells interacted with the 3D nanostructures at rest and after electroporation. We monitored in *real time* different molecular species, such as lipids and proteins, both on the cell membrane and in the cytoplasm. We noticed that there is a characteristic time of 10 min on average, in which the Raman signal increases drastically, that can be related to the opening of nanopores on the cellular membrane. Importantly, during this time window, the 3D plasmonic nanoelectrodes are in contact with the intracellular environment where additional molecular varieties can be investigated, including nucleic acids. The presence of genetic material in the cytosol, together with focus ion beam/scanning electron microscopy (FIB/SEM) cross‐sectioning, suggested poration of the nuclear envelope. This achievement may pave the way to the investigation of nuclear membrane processes as well as controlled intranuclear delivery and sampling. However, it also imposes careful design and exploitation of 3D pillars to prevent potential damage of the nucleus.

## 2 Cells Electroporation and Raman Analysis

**Figure 2:**
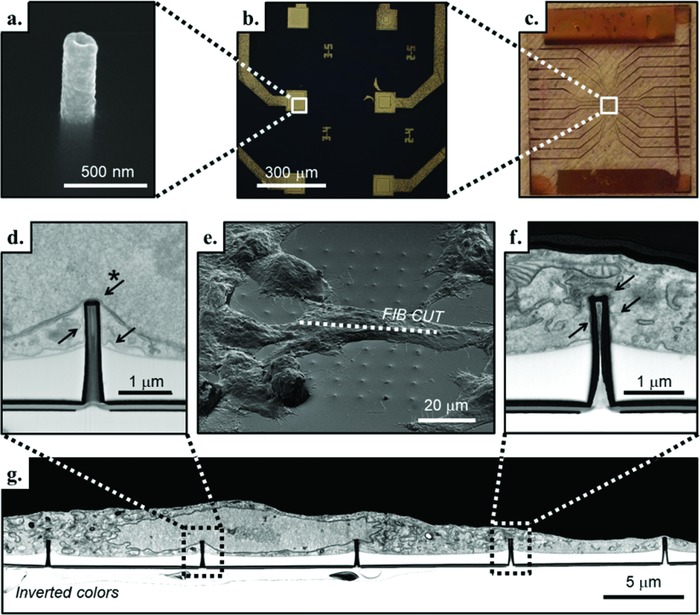
a) SEM image of a single 3D plasmonic nanoelectrode embedded in the SU8 passivation layer. b,c) Magnification of six 3D nanofabricated flat electrodes and the entire MEA‐like device, respectively. e) Tilted SEM image of a fixed and resin‐infiltrated NIH‐3T3 cell cultured on the 3D plasmonic nanoelectrodes. In correspondence with the dotted line, g) the SEM image of the FIB cross section with inverted colors reveals the cell interface with the 3D plasmonic nanoelectrodes. The SU8 passivated flat substrate that is clearly visible (in white, below the cell). d) Inset of the cross section in which the 3D plasmonic nanoelectrode is close to the nuclear envelope (indicated with the starred arrow), and the cell membrane is in tight adhesion with the device (arrows without star). f) Inset of the cross section that shows the plasma membrane tightly wrapped all around the 3D plasmonic nanoelectrode (arrows) and to the flat SU8 passivation layer.

The fabrication process of our device is based on milling by focused ionic beam (FIB) of an optical resist and its consequent inversion.^23^ In contrast to established methods,^21^ this technique allows the fabrication of ordered arrays of 3D nanostructures with a high velocity of milling. Moreover, it is possible to spatially arrange the nanostructures with high precision and to create devices that mix 3D and 2D features, as in MEAs with 3D nanostructures fabricated on each planar electrode.^24^, ^25^ Because of the fabrication process, the 3D nanostructures are hollow. However, being milled on a bulk quartz MEA, the inner nanochannel is closed at the bottom and not through‐hole. We used a device with a MEA‐like configuration consisting of 24 electrodes arranged on a 4 mm^2^ surface. The 3D plasmonic nanostructures fabricated on them were used for both in situ electroporation and SERS spectroscopy (see **Figure** **2**a–c). To have more than one 3D plasmonic nanoelectrode in contact with the same cell, thus enhancing the probability of having contact with a central portion of the cell (for more details, see Figure S5, Supporting Information), the 3D nanostructures were fabricated with a pitch of 10 or 5 µm between each other. The 24 planar electrodes could be addressed independently applying an electrical pulse train, thus porating only the cells under investigation without affecting the rest of the culture.

To avoid cell electroporation from nonspecific sites due to irregularities in gold deposition, the flat surface of the electrodes was passivated leaving only the tips of the 3D nanoelectrodes exposed to the cell culture (see Figure 2a, Experimental Section, and Figure S1, Supporting Information, for more details).^22^ The 3D nanostructure tips also had the highest plasmonic enhancement.^19^

NIH‐3T3 cells were plated at a concentration of 1.5 × 10^4^ cells cm^−2^ and grown for 36 h in controlled conditions. During this time, the cells strongly adhered to the substrate, showing tight sealing with the 3D plasmonic nanoelectrodes (see Figure 2d,f,g and Figure S3, Supporting Information, on the staining and cross‐sectioning procedure), thus allowing the Raman signals coming from the membrane to be enhanced and detected. After 36 h in culture, we replaced the cell medium with phosphate buffer solution (PBS) for performing Raman spectroscopy of the in vitro living cells in liquid. When electroporation is performed, nanopores are created at the interface with the 3D nanoelectrodes, and the cellular membrane begins to settle in an attempt at healing.^26^, ^27^ The capability of the cells to perform mitosis is preserved as well as their viability (see also Figure S2, Supporting Information, for viability tests).^28^

Cells adapt to the substrate and are free to move and replicate because of the short height of the 3D plasmonic nanoelectrodes that protrude from the flat SU8 passivation layer.^28^ The time scale for major displacement of the cells is on the order of hours,^29^ but minor movements are faster, and for this reason, the portion of the plasma membrane in adhesion with the 3D plasmonic nanoelectrodes is not always the same and can change within the experiments. Moreover, each 3D nanoelectrode may be in proximity to a different part of the cell, being closer to the nucleus (inset in Figure 2d) or farther from it, in proximity to a mitochondrion (Figure 2f) or to other organelles. Pioneer works from the last years demonstrated that 3D nanoelectrodes are able to access the intracellular environment for electrophysiological measurements as well as intracellular delivery.^7^, ^21^, ^25^, ^30^ However, as it can be appreciated in Figure 2d, such an approach may have, as a dramatic drawback, the poration of the nuclear envelope. Such an event is a very delicate matter to consider when designing 3D interfaces and devices that work in tight contact with cells and tissues. In fact, it may enable intranuclear delivery of biomolecules and/or sampling of intranuclear content that would be of fundamental importance in many applications. On the other hand, very little is known about potential negative effects of nuclear poration on the overall health and viability of the cells.^31^

While acquiring the SERS signal and using only standard optical microscopy for observing the cells, there is no a priori certain knowledge of the cell portion lying where the measurement is performed. Rather, only a guess can be made of the proximity to the cell nucleus or the edge based on information obtained from the optical images (for more details on the cell positions, see Figure S5, Supporting Information). For our experiments, we relied only on the optical images (see Figure S4c,f, Supporting Information), and the data presented in this study were acquired from 3D plasmonic nanoelectrodes close to the center of the cells. In this configuration, the probability of being in proximity to the nucleus is high, making accessible, in theory, the nuclear envelope and its content.

The experimental procedure consisted of the laser excitation of a single 3D nanoelectrode with a cell lying on it. The Raman excitation was obtained with a monochromatic laser (λ = 785 nm) focused on the 3D plasmonic nanostructure creating intense hot spots in correspondence of its tip. From the literature, it is well‐known that the electromagnetic field of localized plasmonic modes decays very fast (after few tens of nm) in space.^32^, ^33^, ^34^ By characterizing the radial profile of our 3D plasmonic nanoelectrodes, we showed that the optical distribution vanishes within 20 nm from the tip surface, allowing us to detect the Raman signal coming only from a small volume around the 3D nanoelectrode, as shown in Figure S2 (Supporting Information) (more details about the optical distribution can be found in Figure S4, Supporting Information). We performed in situ electroporation by applying a potential difference between the 3D plasmonic nanoelectrode and a reference platinum electrode immersed in the cell culture. The in situ permeabilization in correspondence of the plasmonic hot spots allows the enhancement of the Raman signals where the nanopores are opened, leading to the detection of the changes in the plasma membrane and studying the dynamics of rearrangement of the lipid bilayer. We used the parameters optimized for similar substrates in previous works.^21^, ^22^ In detail, the in situ electroporation was performed using a pulse train with a 20 Hz repetition rate, an amplitude of 3 V (offset at +1.5 V, to have pulses from 0 to 3 V), a pulse length of 100 µs, and a train pulse duration of 10 s. We also acquired Raman spectra with different electroporation parameters. However, the spectra acquired after electroporation with higher or lower voltages did not show significant or valuable information when averaged over several experiments. In fact, higher voltages could lead to bubble formation and to higher degree of permeabilization (bigger nanopores, difficulty in membrane resealing, cell death). Lower applied voltages could be less effective for local permeabilization of the cell membrane.

## 3 Real‐Time Monitoring of Membrane Poration and Intracellular Environment

**Figure 3:**
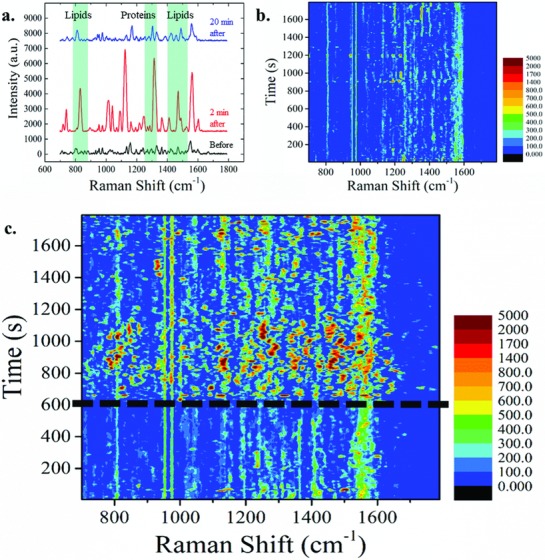
a) Enhanced Raman spectra recorded from the same 3D plasmonic nanoelectrode before (black), 2 min after (red), and 20 min after (blue) the application of the electroporation pulse train. Three regions of the Raman shift are highlighted in which most of the peaks are related to lipids and proteins. The background has been subtracted and the intensities coherently shifted to improve the visualization. Data are from a single experiment. b,c) Colored maps of the average SERS signals of cells lying on top of 3D plasmonic nanoelectrodes excited by a λ = 785 nm laser during 30 min of acquisition. b) The spectra do not show particular features or changes in time when there are no external stimuli applied. c) Average SERS signals of electroporated samples at 10 min from the beginning of the experiments. After electroporation (at *t* = 600 s, marked by the dotted line), new vibrational modes appear, and the average peaks intensities increase. Rapid shifts of new and old peaks occur, meaning that the plasma membrane and the rest of the cell undergo rearrangement. Slowly over time, the signals come back to resemble the signal before the electroporation occurred.

The typical SERS spectrum of a cell lying on the 3D plasmonic nanoelectrode before the electrical pulse train application is shown as a black line in **Figure** **3**a, while the red line represents the typical SERS spectrum acquired from the same Raman electrode just after electroporation, and the blue spectrum is the recorded signal after 20 min from the permeabilization. As can be seen, right after the electroporation we observed a strong increase of the Raman signals. To better highlight this behavior, we monitored the Raman spectra for 30 min with and without electroporation (Figure 3c,b, respectively). In these graphs, the average time‐resolved SERS spectra are presented as color maps (*N* = 6 for the graph in Figure 3b and *N* = 10 cells for the graph in Figure 3c on at least five different cell cultures). For more detail on how the data have been processed, see the Experimental Section and Figure S4 (Supporting Information).

Due to the plasmonic enhancement of the 3D nanoelectrodes, a good signal‐to‐noise ratio was obtained using a time resolution of only 6 s (accumulation of five acquisitions, each lasting 1 se plus the processing time of ≈1 s), reaching a high time detail in respect to the long observation time of 30 min.

Before or in absence of electroporation, the Raman spectra were rather stable, and minor changes appear probably due to the physiological movement of the cell, namely, the natural dynamics of the cell membrane as it moves around the 3D plasmonic nanoelectrode. In contrast, when electroporation was applied (Figure 3c at *t* = 600 s), the signal changed dramatically, showing the appearance of new peaks with much higher intensities than the baseline together with an increase in intensity or temporary disappearance of old peaks. The intensity increase is mainly due to the sudden change in the environment as it became intracellular or partially intracellular, making the cytoplasm and all its content accessible for detection through the nanopores in the cell membrane. By analyzing the spectra in details, we noticed that the majority of changes that appeared after electroporation occurred in the Raman shift regions corresponding to lipids (780–890 and 1400–1550 cm^−1^)^35^ or proteins (1240–1310 cm^−1^).^36^ This is in agreement with the fact that when the plasma membrane undergoes a process of permeabilization, a period of rearrangement of the lipid bilayer follows with the aid of several membrane proteins and protein complexes at the interface with the membrane.^10^, ^27^, ^37^ Additionally, the orientation of the molecules with respect to the electric field can contribute to the peak shifts and the changes in intensity.^38^

These drastic changes in the Raman spectra lasted for ≈10 min, after which the Raman spectra began to settle to the initial values. This behavior can be ascribed to the plasma membrane healing and the resealing of the nanopores that were created through electroporation.^39^, ^40^

After 20 min from application of the electrical pulse train, the Raman peak intensities returned to values comparable to those before the electroporation, and most of the vibrational modes that appeared with the electrical pulses vanished. As this time range is comparable to that found in several other scientific works,^21^, ^26^ this behavior can be associated to the nanopores closure attempts at the interface with the 3D nanoelectrode and to the reformation of the membrane to the pre‐electroporation conditions.

Our analysis aimed to study the averaged behavior rather than a single cell response to the external stimulus, because we could not be sure on what portion of the cell membrane were in contact with the 3D plasmonic nanoelectrode. For the interpretation of the results in fact, we need to keep in mind that live biological systems are governed by extremely complex rules and are affected by many factors, and we assumed that each different region of the plasma membrane would present a slightly different response (see, e.g., Figures S3b and S4a, Supporting Information). In other words, results from single experiments may cause misleading interpretations.

In the following, we provide a more detailed analysis of peaks related to molecule of interest. We followed the time trace of the peaks throughout the experiments to compare their behavior in the presence and in the absence of the electroporating event (for more details on how the peaks were chosen, see the temporal average analysis in Figure S6, Supporting Information).

**Figure 4:**
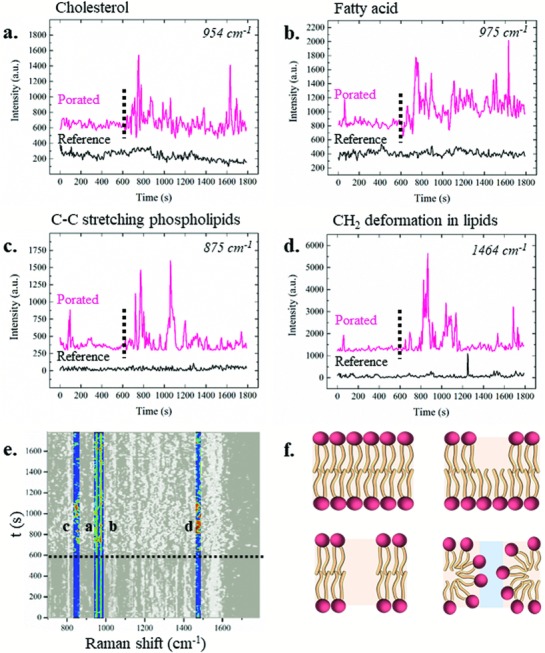
a–d) Average temporal behaviors of lipid related peaks throughout the 30 min of experiments in the absence (black) and in the presence (pink) of the electroporation, which is identified by the dotted line. The peak dynamics are shown on the average spectra. In particular, the dynamics of the a) 954 cm^−1^ peak assigned to cholesterol, b) 975 cm^−1^ peak assigned to fatty acid, c) 875 cm^−1^ peak assigned to the C—C stretching of phospholipids, and d) 1464 cm^−1^ peak assigned to CH_2_\CH_3_ deformations in cholesterol and triacylglycerols. e) Highlighted peaks from the global colored map of electroporated samples. Scale bar from 0 a.u. (black) to 5000 a.u. (red). f) Sketches of possible lipid membrane configurations. Top left: intact lipid bilayer, top right and bottom left: hydrophobic pores in the cell membrane, bottom right: hydrophilic pore.

We noticed that some of the lipid‐related vibrational modes were present throughout the whole measurements, such as the peaks centered at 954 and 975 cm^−1^, assigned, respectively, to cholesterol^19^ and to fatty acid vibrational modes (**Figure** **4**a,b).^35^ Looking to their time trace, the peaks intensities increase with the application of electroporation (pink temporal traces) but only by few hundreds of units with respect to the behavior in the absence of electroporation (black temporal traces). On the other hand, other lipid‐related peaks showed a very different behavior in time after permeabilization of the plasma membrane. Figure 4c,d shows the time trace of the 875 cm^−1^ peak, assigned to the C—C stretching of phospholipids,^35^ and the peak at 1464 cm^−1^ related to CH_2_\CH_3_ deformations in cholesterol and triacylglycerols.^41^ The two peaks presented a dramatic increase in intensity after electroporation, suggesting a drastic change in the presence and orientation in the plasma membrane of the corresponding molecules. In Figure 4e, the peaks represented in the time traces above are highlighted in the average SERS spectra for the sake of clarity. Figure 4f depicts some of the possible configurations of the lipid bilayer. In top left, the cell membrane is sealed, while in the top right and bottom left, the membrane is broken by hydrophobic pores, and in the bottom right, the pore is hydrophilic.^42^

We suggest that these configurations may explain the transient appearance and variations of the lipids peaks right after electroporation. We noticed that the variations reported in Figure 4a–d were not synchronized, suggesting that each molecule had a specific role (or not) in the rearrangement of the membrane after permeabilization in a certain temporal order. Importantly, such a lack of synchronization among the temporal dynamics of the different peaks and the different behavior in respect to the control samples confirmed that the detected changes were not related to the measurement (i.e., laser power oscillations, environment conditions) but only to the electroporation event.

After the membrane reformation, the Raman spectra still present some variations in respect to the initial spectra, possibly due to the membrane fluidity, which is still affected by the electroporation protocol.

### 3.1 Observation of Aromatic Amino Acid and Amide Vibrational Modes

**Figure 5:**
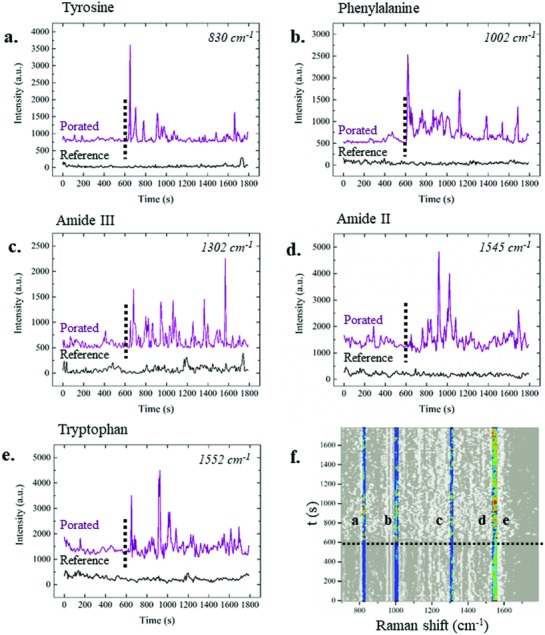
Average temporal behaviors of protein‐related peaks throughout the 30 min of experiments in the absence (black) and in the presence (purple) of electroporation, identified by the dotted line. The peak dynamics are shown on the average spectra. In particular, dynamics of the a) 830 cm^−1^ peak, associated with the tyrosine amino acid, b) 1002 cm^−1^ peak assigned to phenylalanine, c) 1302 cm^−1^ peak that identifies the amide III vibrational mode, d) 1545 cm^−1^ peak assigned to the amide II vibrational mode, and e) 1552 cm^−1^ peak, assigned to the tryptophan amino acid. f) Highlighted peaks from the colored average map. Scale bar from 0 a.u. (black) to 5000 a.u. (red).

The temporal evolution of the protein vibrational modes was studied (**Figure** **5**), and in particular, the dynamics of three peaks assigned to the aromatic amino acids were investigated, including tyrosine, identified at 830 cm^−1^,^43^ the more intense peak at 1002 cm^−1^ related to the phenylalanine,^44^ and the peak at 1552 cm^−1^ assigned to tryptophan.^43^ In addition, the dynamics of the two peaks at 1302 and at 1545 cm^−1^, assigned to the C—N stretching and N—H bending of, respectively, amide III^36^, ^44^, ^45^ and amide II^45^, ^46^ vibrational modes, were studied. Different behaviors were noticed when electroporation was induced (purple lines in Figure 5a–e) relative to measurements made without external stimulation (black spectra in Figure 5a–e).

In particular, the amino acid modes have a very stable and low intensity in the absence of electroporation, while these modes appeared afterward with a very strong intensity (Figure 5a,b,e). In contrast, the amide II and amide III vibrational modes (Figure 5c,d) also presented some intense peaks before the application of the electrical pulse train. However, the effect of the plasma membrane permeabilization was visible in these vibrational modes as well. These dynamics, and in particular the amino acid vibrational modes, suggested the formation of hydrophilic nanopores on the plasma membrane that allowed cytoplasmic proteins to get closer to the 3D plasmonic nanoelectrode, leading to the consequent detection of their enhanced Raman spectra.

The results reported so far show that the use of 3D multifunctional nanostructures combined with MEA and Raman spectroscopy enable following spontaneous physiological changes of the plasma membrane and reorganization processes occurring after electroporation. The presented approach can also give insights into the composition and evolution of intracellular compounds.

### 3.2 Nuclear Poration

From the SEM cross section shown in Figure 2 inset d, one can notice that the nuclear membrane can be in close proximity to the 3D nanoelectrode, thus making nuclear poration feasible. In that scenario, DNA can exit from the safety of the nucleus and move close to the plasmonic enhancer, where its SERS signal can be detected. For this reason, the temporal behavior of the peaks assigned to nucleic acid (DNA and RNA) was analyzed in the presence and in the absence of electroporation.

**Figure 6:**
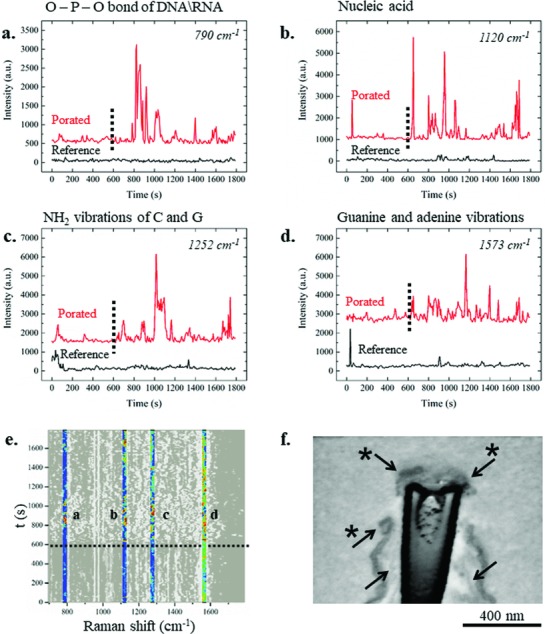
Average dynamic behavior in time of DNA‐ and nucleic acid‐associated peaks. a) 790 cm^−1^ peak associated with the O—P—O stretching of DNA and RNA backbone, b) vibrational mode assigned to nucleic acid at 1120 cm^−1^, c) peak centered at 1252 cm^−1^ associated with vibration of cytosine and guanine, and d) peak at 1573 cm^−1^ related to guanine and adenine vibrational modes. Red spectra are the average of electroporated samples at 600 s (dotted line), while black spectra are the reference to which electroporation has not been applied. e) Highlighted peaks from the global colored map that indicated electroporation. Scale bar from 0 a.u. (black) to 5000 a.u. (red). f) SEM cross section (with inverted colors) of a 3D plasmonic nanoelectrode tip with a cell grown on it. The sample was fixed and analyzed after the application of electroporation. The starred arrows indicate the nuclear envelop, clearly broken close to the edge of the 3D nanostructure. The arrows without the star indicate the plasma membrane.

**Figure** **6**a shows the time evolution of the peak at 790 cm^−1^ that has been assigned to the C′_3_—O—P—O—C′_5_ phosphodiester bond of DNA and RNA,^47^, ^48^ while the vibrational modes at 1120 cm^−1^ have been generically assigned to nucleic acid (Figure 6b).^43^ The peak centered at 1252 cm^−1^ has been assigned to the NH_2_ vibrational modes of cytosine and guanine (Figure 6c),^49^ and the peak at 1573 cm^−1^ has been assigned to guanine and adenine vibrational modes (Figure 6d).^50^ The four Raman enhanced peaks showed a similar behavior in time throughout the experiments, with minimal changes in intensity in the absence of electroporation (black traces and red traces before the dotted line) and larger intensities after application of the electroporating pulse train.

Being able to detect RNA offers a new method to sequence and investigate the whole RNA pool of a single cell within a large culture, offering a new detection method to the challenging field of transcriptomics.^51^, ^52^

Because the Raman signals could also be originated from molecules that are present in the cytoplasm, such as mRNA or ribosomes, we performed a more detailed cross‐sectional SEM investigation of the nuclear membrane after electroporation to corroborate the nuclear poration hypothesis.

Figure 6f shows an SEM cross‐section image of an electroporated cell, suggesting the possibility of directly porating the nuclear envelope to gain access not only to the cytoplasmic compartment, but also to the nuclear environment. In particular, the cross section presents a 3D plasmonic nanoelectrode that was close to the nucleus, and the nuclear membrane appears porated. From the SEM image, it can be seen that the tip of the 3D plasmonic nanoelectrode results in contact with the intranuclear compartment, allowing access to the information stored inside (see also Figure S6, Supporting Information). Thus, although the presented Raman fingerprints cannot be unequivocally attributed to nuclear DNA, the SEM cross section supports the hypothesis that the nuclear content may become accessible for Raman spectroscopy after in situ permeabilization. More experiments are required to investigate this intriguing possibility.

We did not notice any change in the viability of the cells after the electroporation. This result means that, assuming we are in fact assisting to a nuclear poration, the tested experimental conditions are not toxic for the cells.

Interestingly, the protein‐related peaks and the RNA‐ or DNA‐related peaks behaved differently in time. The nucleic acid and backbone vibrations, in fact, appeared a few minutes after electroporation (Figure 6a,c), while the peaks associated with protein vibrational modes presented an increase in their intensity just after application of the electrical pulse train (Figure 5a–c,e). This kind of behavior indicates that the DNA and nucleic acid molecules were not present on the cell membrane, as expected, and they needed some time to diffuse close to the 3D plasmonic nanoelectrodes and finally become detectable after permeabilization of both the plasma and nuclear membranes.

In general, these results suggest a variety of different membrane reforming mechanisms because of the different recruitment times of different molecules.

## 4 Conclusion

In this work, we presented a multifunctional platform based on MEA refined with 3D plasmonic nanostructures, which acts both as a plasmonic enhancer and as nanoelectrodes, allowing local permeabilization of the cell membrane exactly in correspondence with the plasmonic hot spot. Using enhanced Raman spectroscopy, we studied the local permeabilization of the cellular membrane and recorded the fingerprints of the molecules involved in the subsequent rearrangement for several minutes. The results of the molecular rearrangement following permeabilization of the cell membrane are in accordance with data in the literature. In fact, the experiments suggested an average closure time of the nanopores on the order of 10 min after electroporation,^39^, ^40^ followed by a settlement period in which the membrane still results more fluid than it was before the electroporation. Remarkably, during this time window, the 3D nanoelectrode was in direct contact with the cytosol, thus providing insight into the intracellular compounds in close proximity to the 3D plasmonic nanoelectrode. Raman peaks related to lipids, proteins, and nucleic acids can be monitored. Surprisingly, DNA‐related peaks could also be found, thus suggesting nuclear envelope poration. The latter was supported by SEM cross sections, and it may represent a straightforward approach to sample or to deliver biomolecules into the nucleus. The presented method can also be a powerful approach to investigate how cells behave or proliferate on 3D nanostructures, which are promising substrates for next‐generation tissue engineering and prostheses.

## 5 Experimental Section

*Fabrication and Passivation*: MEAs were fabricated by standard lithographic methods on quartz wafers. 3D vertical nanostructures were fabricated by milling through an optical resist by means of FIB. The device was passivated with an epoxy polymer (SU8), leaving only the tip of the 3D nanostructures exposed to the environment. More details on the fabrication are in the Supporting Information.

*Cell Culture*: Before seeding of the cells, the devices were treated with plasma oxygen (60 s, 100% O_2_, 100 W) to improve their wettability and sterilized through UV exposure (20 min) in a laminar flow hood. NIH‐3T3 cells were seeded on the devices with (1.5 × 10^4^ cells cm^−2^) in Dulbecco's modified Eagle's medium (DMEM) cellular medium with penicillin/streptomycin (1% pen/strep) antibiotic and fetal bovine serum (10% FBS, all by Sigma‐Aldrich) and grown at controlled humidity, CO_2_ concentration, and temperature for 36 h prior to performing experiments.

*Cell Staining, FIB Cross Section, and SEM Imaging*: Cells were fixed with glutaraldehyde solution (2.5%) in Na cacodylate buffer (0.1 m) for at least 1 h on ice. Samples were extensively washed in buffer solution and incubated with glycine (20 × 10^−3^ m) in buffer solution on ice. Then, a recently developed RO‐T‐O staining protocol was performed,^53^ and samples were embedded in a thin layer of Spurr resin. For more details on the procedure, see the Supporting Information. The cell cross sections were performed using a dual‐beam Helios Nanolab 650 by ThermoFisher. Slicing of the specimens was performed using high ionic currents (9.3 or 0.79 nA) after Pt deposition using the gas injection system (GIS) of the instrument. More details can be found in the Supporting Information. Imaging was performed with the samples tilted at 52° with respect to the electron beam, and backscattered electrons were collected using a TLD detector in immersion mode. The acceleration of the primary electrons was 3 kV, and the current used in the imaging process was *i* = 0.40 nA. The cross‐sectioned images are presented with inverted colors to emphasize the cellular membranes.

*Electromagnetic Field Enhancement Simulation*: A finite element method analysis implemented in COMSOL Multiphysics software was performed, by simulating a 1.8 µm high 3D nanoelectrode covered with 60 nm of gold and embedded in SU‐8 polymer, leaving 700 nm of 3D plasmonic nanostructure exposed to the interface with water. The incident light is a monochromatic (λ = 785 nm) linearly polarized plane wave.

*Electroporation and Raman Measurement*: In situ electroporation was performed by applying a pulse train with a 20 Hz repetition rate, an amplitude of 3 V, a pulse length of 100 µs, and a train pulse duration of 10 s. Raman spectra were measured by a Renishaw inVia Raman spectrometer with a Nikon 60× water immersion objective and a 1.0 NA delivering a 785 nm laser with a power of ≈0.6 mW. Each spectrum was the result of 1 s acquisition accumulated five times.

*Data Analysis*: All of the Raman signals acquired during laboratory activities were processed with custom‐made programs in MATLAB 2017 or C++, and then visualized in Origin 9.0 prior to a second analysis using this software. The data acquired by Wire3.4 Renishaw software were exported and saved in .txt format in order to facilitate their transfer. For more details, see the Supporting Information.

## Conflict of Interest

The authors declare no conflict of interest.

## Supporting information

Supplementary
