# Supplementary material for: Enhanced Raman Investigation of Cell Membrane and Intracellular Compounds by 3D Plasmonic Nanoelectrode Arrays
Source: Adv Sci (Weinh). 2018 Oct 23;5(12):1800560. doi: 10.1002/advs.201800560 (PMC6299714; doi:10.1002/advs.201800560)
Supplement: Supplementary file 1 — Supplementary [file ADVS-5-1800560-s001.pdf]

Copyright WILEY-VCH Verlag GmbH & Co. KGaA, 69469 Weinheim, Germany, 2018.

Supporting Information

for *Adv. Sci.,* DOI: 10.1002/advs.201800560

Enhanced Raman Investigation of Cell Membrane and Intracellular Compounds by 3D Plasmonic Nanoelectrode Arrays

*Valeria Caprettini, Jian-An Huang, Fabio Moia, Andrea Jacassi, Carlo Andrea Gonano, Nicolò Maccaferri, Rosario Capozza, Michele Dipalo, and FrancescoDe Angelis**

Copyright WILEY-VCH Verlag GmbH & Co. KGaA, 69469 Weinheim, Germany, 2016.

Supporting Information

Enhanced Raman investigation of cell membrane and intracellular compounds by 3D plasmonic nanoelectrode arrays

*V. Caprettini, J.A. Huang, F. Moia, A. Jacassi, C. A. Gonano, N. Maccaferri, R. Capozza, M. Dipalo, F. De Angelis**

Contents

SI 1. Fabrication and passivation 1
SI 2. Viability tests 2
SI 3. Cell staining and FIB cross section 2
SI 4. Electromagnetic field distribution on the 3D plasmonic nanoelectrode 3
SI 5. Data analysis and background subtraction 4
SI 6. Influence of cell position on the Raman spectra 6
SI 7. Temporal average 7
SI 8. Nuclear Poration 8

SI 1. Fabrication and passivation

On a standard quartz MEA, 24 gold electrodes are opened in a central area of the device, each of which has an exposed area of 60 × 60 μm^2 and a center-to-center inter-electrode space of 400 μm. An optical resist (S1813, by Shipley) has been spin-coated on top of the MEA and a sacrificial layer of gold has been sputtered on top of it. The fabrication process of the 3D plasmonic nanoelectrodes consists in the focus ion beam (Helios Nanolab 650, by Thermofisher) milling of the polymer and its inversion by the secondary electrons. The gold sacrificial layer is wet etched, and the unexposed resist developed by acetone and isopropanol washing. Plasma ashing allows for a final cleaning of the unexposed polymer. Gold is deposited on the 3D nanostructures to make them plasmonic and electrically conductive.[1],[2]
The 3D nanostructured MEAs have been passivated with an epoxy polymer layer (SU8), in order to leave exposed to the cellular culture and electrically active only the tips of the 3D plasmonic nanoelectrodes. Briefly, the SU8 polymer is spin-coated on to the device with a final height that matches the one of the 3D nanoelectrodes, inverted by UV exposure, post-

baked at 95°C; finally, a plasma etching procedure has been implemented so to leave only 700 nm of the 3D plasmonic nanoelectrode exposed to the surrounding environment (200 W, 30% O_2, 90 sec).[3]

SI 2. Viability tests

To assess the viability of the cells on our device, live-dead assays have been carried out. Calcein AM and Propidium Iodide have been added to the cell culture at a concentration of 2 μM and incubated at room temperature for 20 minutes to allow the Calcein AM to be internalized and processed by the cells. The results are shown in Figure S1.

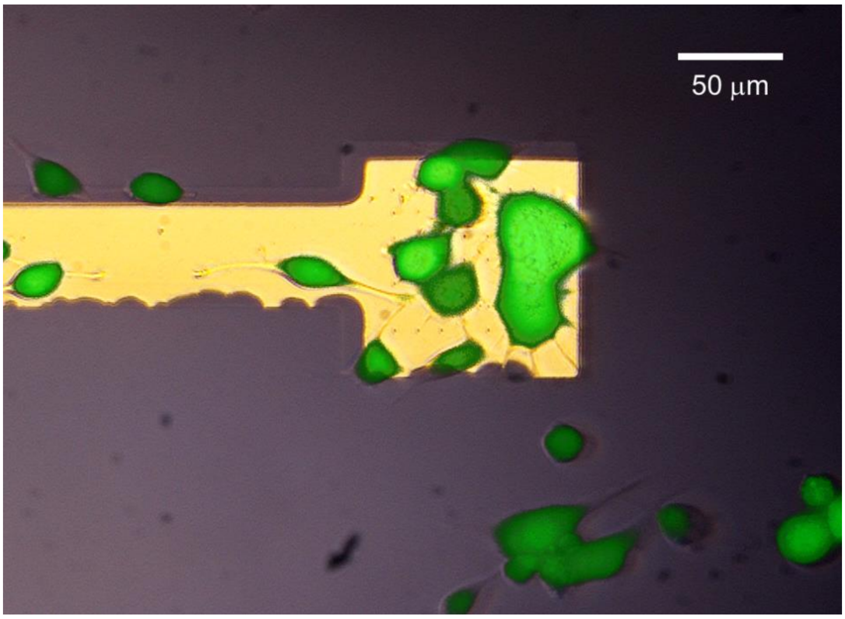

Figure S1. *Live dead essay on cells cultured on the 3D plasmonic nanoelectrodes fabricated on flat microelectrodes.*

SI 3. Cell staining and FIB cross section

Cells have been fixed with a 2.5% glutaraldehyde solution in 0.1 M Na cacodylate buffer for at least 1 hour on ice. Samples have been extensively washed in buffer solution, incubated with 20mM glycine in buffer solution on ice and a recently developed RO – T – O staining protocol has been performed.[4] Thereafter, samples have been incubated in 5% uranyl acetate in aqueous solution overnight at 4°C, and a last step in tannic acid (0.15% in milliQ) have been performed to diminish the background noise. Dehydration is performed exchanging the water with increasing concentration of ethanol until all the water has been substituted. Samples have been embedded in Spurr resin increasing its concentration in ethanol buffer. Upon completing the infiltration, the resin excess is washed away by gently rinsing pure

ethanol on the samples while they are kept vertical to help the resin draining, and the epoxy polymer is cured at 65°C in oven overnight.

The cross sections of the cells are performed using a dual beam Helios Nanolab 650 by ThermoFisher. Samples are metallized, glued to a standard stub with conductive silver paste, and a protective layer of platinum is deposited prior the slicing of the cross sections by beam assisted deposition. The trenches are milled using an acceleration of 30 kV and a high ionic current of i = 9.3 nA; a polishing cross section is milled using a lower beam current of i = 0.79 nA.

SI 4. Electromagnetic field distribution on the 3D plasmonic nanoelectrode

We simulated with a finite element method implemented in Comsol Multiphysics software, the electromagnetic field intensity distribution for an isolated 3D plasmonic nanoelectrode. As it can be clearly seen from Figure S2, hot-spots in contact with the environment, and so with the cell membrane, are created on the tip of the 3D nanostructure. We analyzed the field distribution along the pillar direction and along the direction of the polarization of the applied electromagnetic field, viz. a monochromatic (λ = 785 nm) linearly polarized plane wave. As it can be inferred by looking the field intensity distribution as a function of the distance from the 3D plasmonic nanoelectrode surface, the Raman intensity (that is proportional to E^4) decays very rapidly after 20 nm, in according with a very well-known behavior of the electromagnetic field decay of localized plasmonic modes.[5,6] Some local roughness can modify this number and a more conservative estimate is between 15 and 30 nm. For this reason, once we porated the cell membrane we can state that our sensitivity is of the order of tens of nanometers close to the hot-spots region, more precisely we calculated our signal to be generated within a range of maximum 30 nm from the antenna surface.

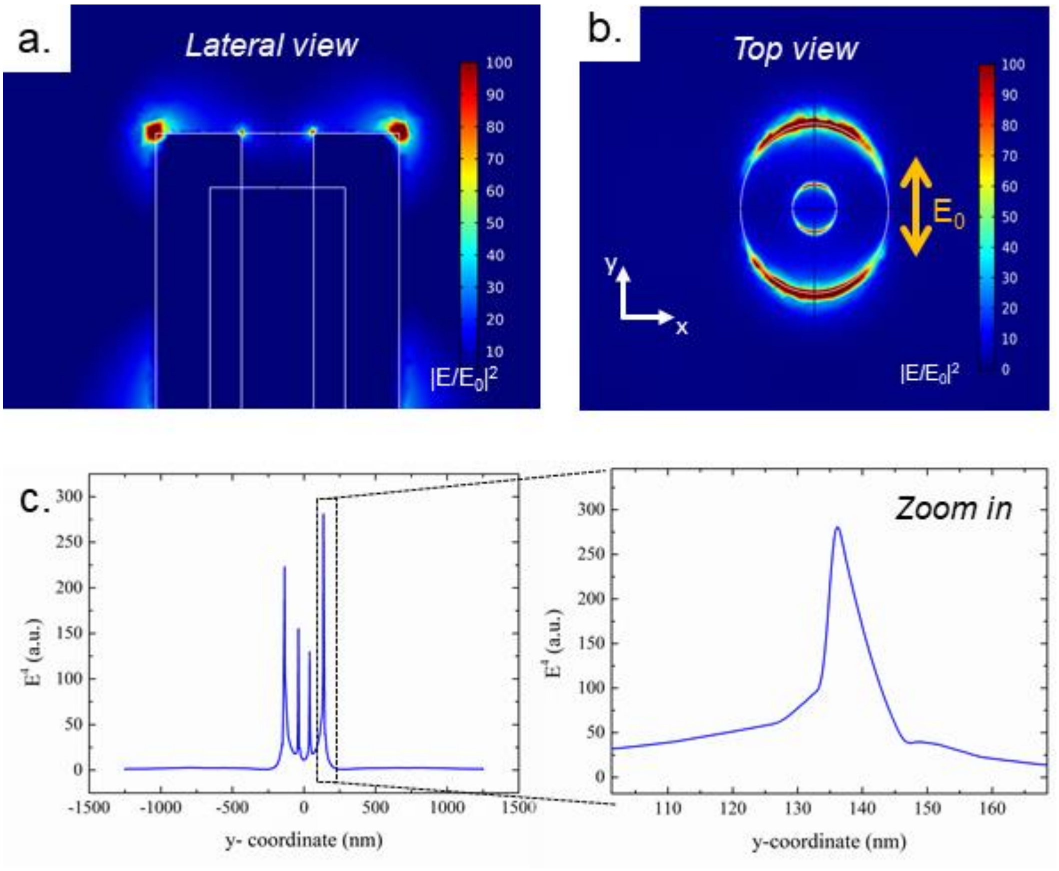

Figure S2. *Electromagnetic field intensity distribution around the 3D plasmonic nanoelectrode. a) z-profile of the electromagnetic intensity ($|E/E0|^2$) field distribution of the 3D plasmonic nanoelectrode tip immersed in water. b) Electromagnetic intensity field distribution profile of the hot-spots exposed to the water environment in the x-y plane. c) E^4 intensity distribution profile along y-direction at the hot spots central position (along z-direction) with zoom in that shows the decay within 20 nm from the 3D nanoelectrode surface.*

SI 5. Data analysis and background subtraction

To make all the experiments comparable within each other, a temporal interpolation was carried out using the built-in *interp2* MATLAB 2017 function. This function have a double use, since it allow the automatic removal of isolated Raman peaks due to cosmic rays interaction with the setup. In fact, the *interp2* function average each value with the adjacent values in time.

The time resolved Raman spectra from different experiments have been averaged together to study a collective behavior of the molecular dynamics of the cell membrane in presence of *in situ* electroporation on the 3D nanostructures.

The average intensity of the Raman spectra for each time instant t_i and every wavenumber R_j, has been calculated as

$$\langle I\rangle(t_{i,}R_j) = \frac{1}{N_s}\sum_{s=1}^{N_s} I_s(t_{i,}R_j)$$

where N_S represents the number of experiments over which the average has been calculated.

The background of the peaks has been substracted from the measurements after its evaluation. The background estimation algorithm is written in C++ programming language, adopting graphic and analytical libraries of Cern Root Data Analysis Framework. In particular, the TSpectrum Class Reference is used as Advanced Spectra Processing routine. This class contains advanced spectra processing functions for:

– One and two-dimensional background estimation
– One and two-dimensional smoothing
– One and two-dimensional deconvolution
– One and two-dimensional peak search

This function calculates background spectrum from source spectrum. The function allows to separate useless spectrum information (continuous background) from peaks, based on Sensitive Nonlinear Iterative Peak Clipping Algorithm. The sensitive nonlinear peak clipping algorithm is the basis of the methods for estimation of the background in multidimensional spectra, leaving the signal intact.

Before the subtraction of the background, all the Raman experiments presented a broad fluorescent band between 1100 and 1700 cm^{-1}, as shown in the color map of the single Raman experiment presented in Figure S3 a that covers the peaks shifts. After the background subtraction (Figure S3 b) only the peaks remain, allowing an easier reading of the data.

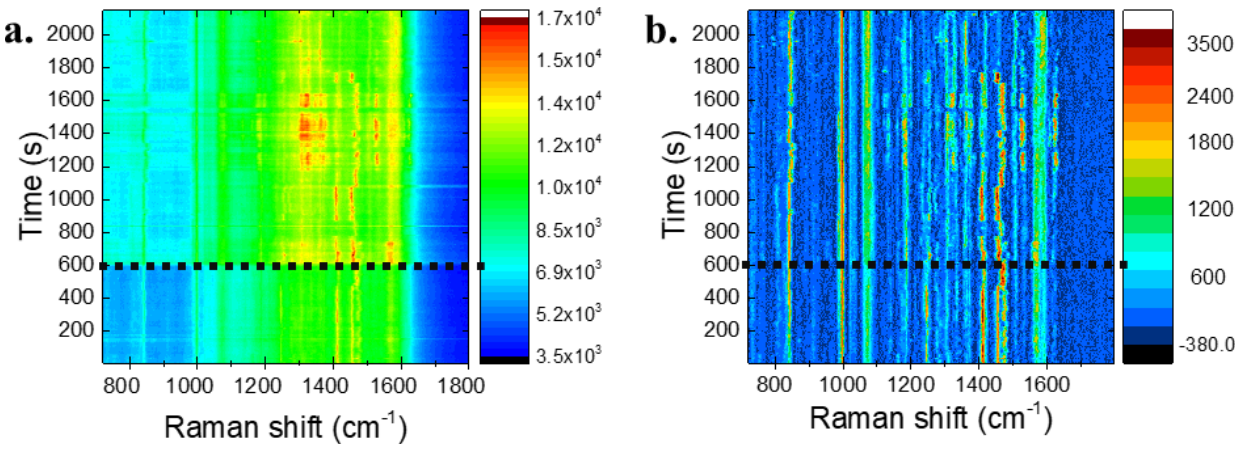

Figure S3. *a) Color map of a single experiment before the background subtraction. Electroporation has been performed at 600 sec. Between 1100 and 1650 cm^{-1} a broad fluorescent peak makes hard to recognize the Raman peaks. b) Color map of the same experiment after the background subtraction.*

SI 6. Influence of cell position on the Raman spectra

Some of the experiments have shown a loss in the signal after the application of the electroporation, while other spectra exhibited an increase in the signal intensity; this effect seems to be associated to the different position of the cell on the 3D nanoelectrodes. The following graphs are color maps of time-resolved measurement over 35 minutes of acquisition. On the x-axis the wavenumber *k*, or Raman shift in cm^{-1} is represented, while on the y-axis there is the time. The different colors are different intensities of the signal, from the low intense in blue, to the most intense in red/dark red.

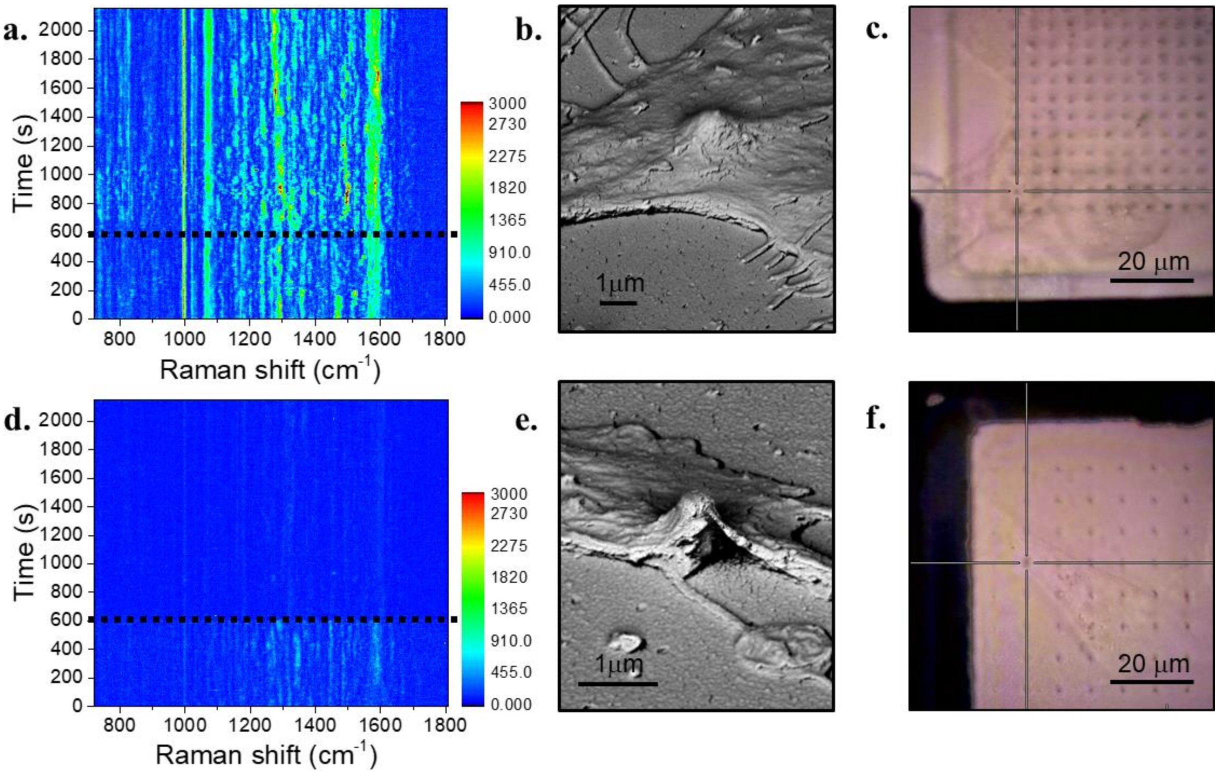

Figure S4. *a) Color map of the time-resolved Raman enhanced acquisition over 35 minutes of time coming from a 3D nanoelectrode in contact with a central part of the cell membrane. After 10 minutes, the electroporation protocol is applied (dotted line); the signal increases in intensity, new peaks appear and other start to shift in wavenumber. b) SEM image and c) optical image of 3D nanoelectrode with a cell on top of it tightly wrapped. The cell membrane is also in adhesion with the flat substrate surrounding the 3D nanoelectrode. d) Time-resolved Raman enhanced acquisition over 35 minutes of time coming from a 3D nanoelectrode in contact with a cell edge. The initial spectrum is lower in intensity respect to the previous; after 10 minutes electroporation protocol is applied (dotted line) and the signal disappears and do not recover in time as if the cell moved away from the 3D nanoelectrode. e) SEM image and f) optical image of a 3D nanoelectrode in contact with a cell edge. The cell membrane is not tightly sealed all around the 3D nanostructure.*

It has been noticed that when the plasma membrane is wrapped around the 3D nanoelectrode and is also tightly attached to the flat substrate around it, on average the signal following the

electroporation increases in intensity (see Figure S4 a, b, c). On the contrary, when the tight sealing between the 3D nanoelectrode and the cell is limited only to a part of the membrane, the initial spectrum results to be lower in intensity and an electroporating event will cause the loss of the signal with no recovery in time (see Figure S4 d, e, f). The best hypothesis to explain these behaviors is that, if the membrane is *floating* on the 3D nanoelectrodes, *in situ* electroporation will cause the membrane to detach from the 3D plasmonic hot-spot. On the contrary, when the cell is tightly adherent around the 3D nanoelectrode, an electrical pulse train will increase the adhesion tightness.

After observing this phenomenon several times, we decided to focus only on Raman signals acquired in a central region of the cell, avoiding the recordings coming from 3D plasmonic nanoelectrodes on the edge of cells.

SI 7. Temporal average

An average in time has been calculated to highlight which peaks have shown changes in time (appearance or disappearance) after the electroporation and different binning times have been evaluated. In the end, an average over the first 10 minutes (*preporation* time, from t = 0 sec to t = 594 sec) has been compared to bins of 5 minutes after the electroporating event (*postporation* time). The acquisitions between 594 sec and 612 sec have been discarded to avoid the influence of the electrical pulse train.

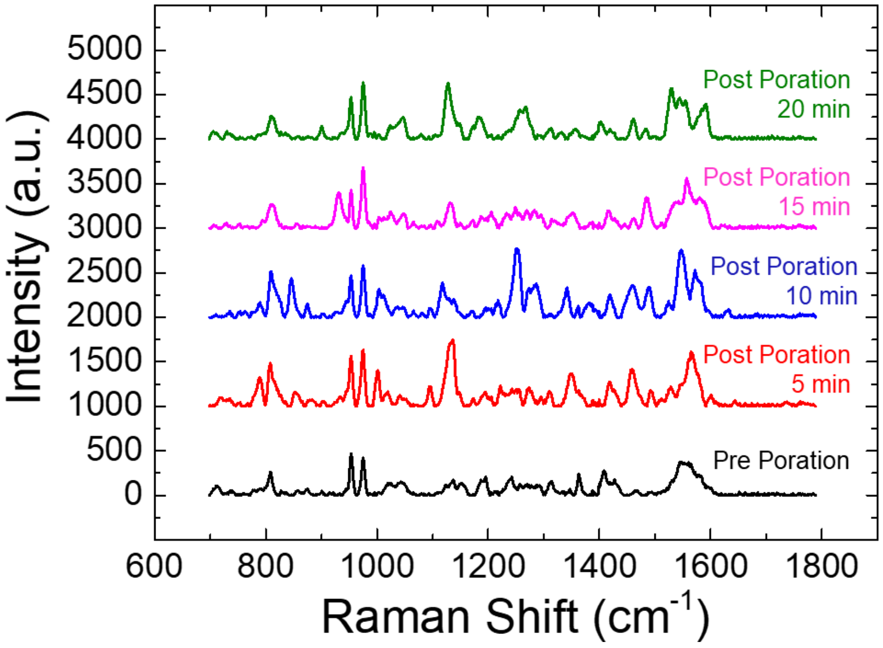

Figure S5. *Temporal analysis of the averaged Raman signals. The black spectrum is the average of the pre-electroporation signals, from t = 0 sec to t = 594 sec. The others Raman spectra are the averages of different time slots after the electroporation, in particular the average of the Raman signals*

between t = 612 sec and t = 900 sec (red line), the average of the Raman spectra from t = 900 sec to 1200 sec (blue spectrum), from t =1200 sec and 1500 sec (pink spectrum) and from t = 1500 sec to t = 1800 sec (green line).

The temporal average has been calculated over the total averaged Raman-signal (Figure 3c of the main text) and the five relatives spectra are shown in Figure S5. Each spectrum has been shifted in intensity to ease the visualization. The black spectrum is the average of the first 10 minutes of the experiments, before the electroporation has been applied. The red spectrum is the average of the aquired spectra from 612 sec and 900 sec, the blue spectrum is the average of the measurements from 900 sec and 1200 sec. The pink spectrum is the average of the measurements performed from 10 minutes after the electroporation and 15 minutes after the electroporation (1200 sec and 1500 sec), while the green spectrum is the average of the spectra acquired in the last 5 minutes of recordings (from 1500 sec and 1800 sec).

SI 8. Nuclear Poration

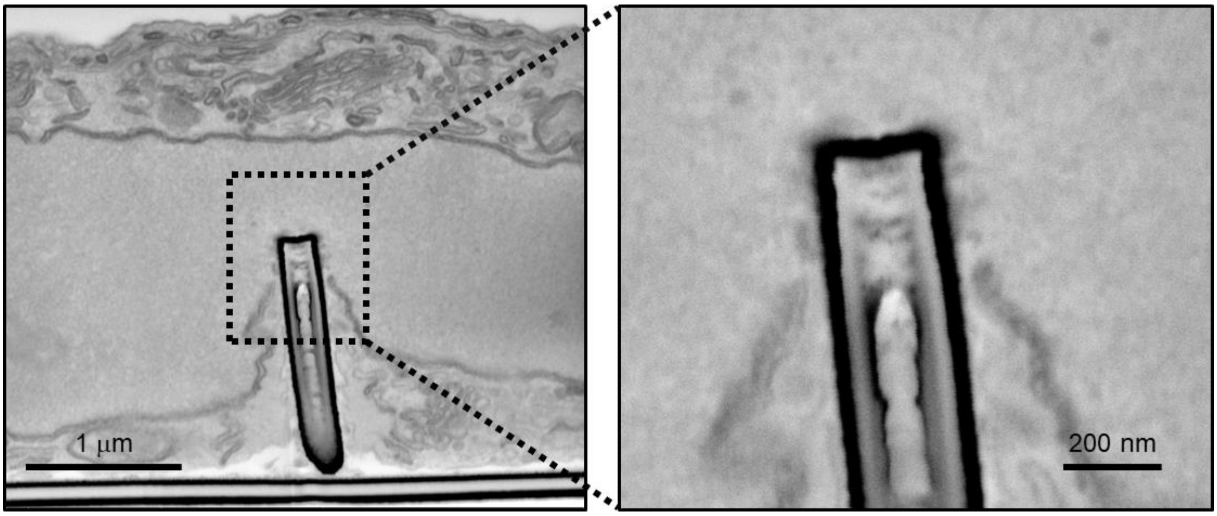

Figure S6. *SEM cross section with inverted colors of a 3D nanoelectrode embedded in a cell after electroporation, with inset showing the magnification at the tip. The 3D nanoelectrode tip is in close proximity to the nucleus, and nanopores are present on the nuclear envelop following the electrical stimulus.*

References

[1] F. De Angelis, M. Malerba, M. Patrini, E. Miele, G. Das, A. Toma, R. P. Zaccaria, E. Di Fabrizio, *Nano Lett.* **2013**, *13*, 3553.

[2] M. Dipalo, G. C. Messina, H. Amin, R. La Rocca, V. Shalabaeva, A. Simi, A. Maccione, P. Zilio, L. Berdondini, F. De Angelis, *Nanoscale* **2015**, *7*, 3703.

[3] V. Caprettini, A. Cerea, G. Melle, L. Lovato, R. Capozza, J.-A. Huang, F. Tantussi, M. Dipalo, F. De Angelis, *Sci. Rep.* **2017**, *7*, 8524.

[4] A. Belu, J. Schnitker, S. Bertazzo, E. Neumann, D. Mayer, A. OffenhÄusser, F. Santoro, *J. Microsc.* **2016**, *263*, 78.

[5] N. Maccaferri, K. E. Gregorczyk, T. V. A. G. de Oliveira, M. Kataja, S. van Dijken, Z. Pirzadeh, A. Dmitriev, J. Åkerman, M. Knez, P. Vavassori, *Nat. Commun.* **2015**, *6*, 1.

[6] J. A. Huang, Y. Q. Zhao, X. J. Zhang, L. F. He, T. L. Wong, Y. S. Chui, W. J. Zhang, S. T. Lee, *Nano Lett.* **2013**, *13*, 5039.
